# Conjuring cognition: a review of educational magic-based interventions

**DOI:** 10.7717/peerj.8747

**Published:** 2020-03-09

**Authors:** Richard Wiseman, Caroline Watt

**Affiliations:** 1 School of Psychology and Sport Science, University of Hertfordshire, Hatfield, Hertfordshire, United Kingdom; 2 School of Philosophy, Psychology and Language Sciences, University of Edinburgh, Edinburgh, Midlothian, United Kingdom

**Keywords:** Magic, Psychology, Education, Cognition, Belief, Science, Critical thinking, Conjuring, Pedagogic

## Abstract

For hundreds of years, magic tricks have been employed within a variety of pedagogic contexts, including promoting science and mathematics, delivering educational messaging, enhancing scepticism about the paranormal, and boosting creative thinking for product design. This review examines this diverse body of work, focusing on studies that have assessed the impact of such interventions. Although the studies tended to yield positive outcomes, much of the work suffered from methodological shortcomings, including measuring the impact of interventions over a relatively short period of time, focusing on self-report measures and failing to employ control groups. The paper makes several recommendations for future study in the area, including assessing the longer-term impact of magic-based interventions, comparing these interventions to other types of pedagogic techniques, focussing on knowledge retention and behavioural outcomes, and collaborating with magicians to develop more impactful interventions.

## Introduction

Around the turn of the last century, several psychologists started to examine the stratagems used by magicians to fool their audiences (e.g., Binet, 1894; Jastrow, 1896; Triplett, 1900). Over the years, researchers have continued to explore the topic (e.g., Bernhard, 1936; Randal, 1982; Lamont & Wiseman, 1999; Schiffman, 2005), with the area recently receiving increased attention (e.g.,  Kuhn, Amlani & Rensink, 2008; Martinez-Conde, Macknik & Blakeslee, 2010).

One aspect of this work has explored whether observing or performing magic tricks can help to promote wellbeing by, for instance, increasing self-esteem, improving motor skills and enhancing mood (for reviews, see Lam, Lam & Chawla, 2017; Wiseman & Watt, 2018; Bagienski & Kuhn, 2019). In general, these studies have obtained positive findings, and suggest that magic-based interventions have the potential to act as an effective and economical tool for promoting physical, emotional and psychological wellbeing.

For hundreds of years a small number of magicians, psychologists, educational practitioners and researchers have also explored ways in which magic tricks can be employed within a variety of pedagogical contexts. Rather than focusing on issues related to wellbeing, this work has focused on a range of more cognitively oriented factors, such as critical thinking and science education.

There are several reasons to believe that magic tricks can act as the basis for effective, and possibly unique, forms of educational interventions. On a cognitive and emotional level, magic tricks possess many of the attributes associated with desirable pedagogic interventions, namely the potential to provoke a sense of curiosity, surprise and interest (Parris et al., 2009; Subbotsky, 2010; Rensink & Kuhn, 2015; Leddington, 2016). On a social level, they often involve several desirable attributes, including face-to-face interaction, clear communication and a sense of collaboration. On a practical level, magic appeals to both children and adults alike, and many tricks are economical to stage because they can be performed with everyday objects. Finally, unlike many arts-based activities (such as learning a musical instrument or dance), many magic tricks can be learnt in a surprisingly short period of time and so quickly give a sense of progress and mastery.

However, despite these advantages, work exploring the educational benefits of magic is little known within mainstream academia, in part because it has frequently been presented in relatively obscure publications. This paper brings together this diverse body of work, reviews research that has assessed the impact of such magic-based interventions, and makes recommendations for future work in the area.

## Survey Methodology

English-language literature relating to the use of magic tricks within an educational setting was identified by (i) individually entering the search terms ‘magic’, ‘education’, ‘serious fun’ and ‘learning’ into databases from both academia and the magic community (including Google Scholar, Ovid Medline, Scopus and ‘Ask Alexander’ from the Conjuring Arts Research Centre), (ii) drawing on existing knowledge within academia and magic, (iii) searching key books and articles that explore the relationship between magic, psychology and science (including Lamont & Wiseman, 1999; Rensink & Kuhn, 2015; Lam, Lam & Chawla, 2017; Wiseman & Watt, 2018; Bagienski & Kuhn, 2019; Kuhn, 2019), (iv) examining the material referenced in located papers and books, and (v) contacting the authors of this material for additional information. All searches were undertaken in September 2019.

In line with Wiseman & Watt’s (2018) work examining how magic tricks can be used to boost wellbeing, this review focused on material that reported some type of qualitative and/or quantitative evaluation. In addition, it concentrated on material reported in books and journal articles, and excluded unpublished reports and theses (see, e.g., Elder et al., 2012; Crossman, 2013; Ogren, 2014). The resulting literature appeared across a diverse range of disciplines, and was classified into four main areas: promoting science and mathematics, delivering information and educational messaging, enhancing scepticism about the paranormal, and boosting creative thinking for product design. This review will assess each of these areas in turn.

### Promoting science and mathematics

Presenting scientific demonstrations within the context of magic tricks has a very long history. The first century inventor and engineer ‘Hero of Alexandria’ described how a variety of scientific phenomena could be presented as conjuring, such as jugs that poured water or wine on command, and a trumpet that mysteriously sounded when temple doors were opened (Woodcroft, 1971). This trend for framing science demonstrations within the context of conjuring continued through the centuries (for a review see Dawes, 1979; Lachapelle, 2008) and became especially popular during the 1800s (e.g., Brewster, 1832; Pepper, 1890). The area still attracts a considerable amount of interest, with modern-day writers producing ‘science magic’ books for the general public (e.g.,  Gardner, 1941; Gibson, 1975; Windley, 1976; Friedhoffer, 1990; Spangler, 2010), and academics publishing journal articles on the topic (e.g.,  Subramaniam & Toh, 2004; Ruiz, 2006; Featonby, 2010; Ellenstein, 2017). In related work, Österblom et al. (2015) have noted how an understanding of the way in which magic tricks exploit perceptual and cognitive biases might help those wishing to devise and conduct scientific studies.

Similarly, over the centuries writers have frequently presented mathematical principles within the guise of magic tricks (see, e.g., Van Etten, 1633; Badcock, 1818; Ball, 1892; Heeffer, 2006; Matsuyama, 2016), with recent work in the area including both book-length treatments of the topic (e.g.,  Gardner, 1961; Fulves, 2003; Diaconis & Graham, 2012; Mulcahy, 2013; Benjamin, 2015) and journal articles (e.g.,  Amir-Moez, 1965; Koirala & Goodwin, 2000; Simonson & Holm, 2003; Matthews, 2008; Lesser & Glickman, 2009). In related work, researchers have also used mathematics-based magic tricks to help communicate key ideas in computing, including algorithms, binary theory and data structures (e.g.,  Bell et al., 2009; Garcia & Ginat, 2012; Garcia & Ginat, 2016; Greenberg & Reed, 2018; Hilas & Politis, 2014; Ferreira & Mendes, 2014; Way, 2007). Some of the most comprehensive work in this area has been carried out as part of the ‘cs4fn’ (‘Computer Science for Fun’) initiative at Queen Mary University of London, and has involved creating downloadable e-books, a specialist website and several live shows (Curzon, McOwan & Black, 2009).

Several researchers have examined the impact of using magic tricks to promote science and mathematics, with much of the work in this area using qualitative methods to assess students’ interest in science.

For instance, Papalaskari et al. (2006) ran a two-week summer school in which teenagers helped to stage a one-day ‘science magic’ event for 6 to 12 year old children. A qualitative assessment of the project suggested that the teenagers found the event enjoyable and exciting, but that some of the younger children failed to appreciate the science behind the magic. The organizers restaged the event and added a booklet that emphasized the science involved in the demonstrations (Papalaskari et al., 2007). A qualitative evaluation of this latter event suggested that the teenagers had become more interested in science as a result of their participation.

Similarly, Bagnoli, Guarino & Pacini (2019) delivered ‘science magic’ lectures to 15 to 20 year old students, with subsequent surveys and interviews suggesting that the lectures were positively received, had acted as a catalyst for additional study, and that some students had re-staged the demonstrations in their own time. Researchers involved in the ‘cs4fn’ initiative staged live ‘computing magic’ shows for teenage students, with feedback indicating that the students had found the shows enjoyable and had an increased understanding of key concepts (Curzon & McOwan, 2008; Curzon, McOwan & Black, 2009). Finally, Ferreira & Mendes (2014) had undergraduate students assess a lecture containing mathematical card tricks, with the results suggesting that the session was engaging and motivating, and that the tricks had boosted understanding.

Although these results appear encouraging, these studies had no control groups, and employed self-report measures of understanding rather than knowledge-based tests. However, three additional studies have adopted more experimental procedures and assessed knowledge acquisition.

First, Lin et al. (2014) and Lin et al. (2017) staged physics lessons with and without ‘science magic’ demonstrations to two groups of 13 to 14-year-old students. Data from both ‘The Science Attitude Scale’ (Lin, 2009) and qualitative assessments suggested that the demonstrations boosted the students’ awareness about science, improved their enjoyment of the lecture and enhanced their self-confidence in learning science. Importantly, the students who had observed the ‘science magic’ demonstrations also obtained significantly higher scores on a multiple-choice test about the content of the lesson.

Second, Taufiq, Suhandi & Liliawati (2017) staged physics lessons with and without a ‘science magic’ demonstration to two groups of 13–14-year-old students. Those who saw the demonstration obtained significantly higher scores on a subsequent test about the content of the lesson.

Finally, Hilas & Politis (2014) assessed the impact of incorporating magic-based interventions into computer science lectures for two cohorts of college students. Survey feedback was compared to data obtained during a previous seminar and showed that students rated the course as significantly more enjoyable, interactive and stimulating. There was no significant improvement in the course pass rate. However, the ‘science magic’ demonstrations were delivered alongside several other interventions (including drama and games), and thus it’s difficult to isolate the impact of the magic tricks.

Future work in this area would benefit from moving beyond self-report measures of enjoyment and engagement, and focusing more on behavioural-oriented outcomes, such as whether science-based magic tricks boost self-motivated learning and activities. In addition, the work would also benefit from the use of control and comparison groups, and using knowledge-based tests of understanding and information retention. This work could explore a range of variables, including the impact of magic-based demonstrations compared to non-magic-based demonstrations, live demonstrations versus those presented on video, and demonstrations performed by educational practitioners compared to those carried out by students. This work has the potential to explore and inform a range of topics within cognition and neuropsychology, including whether hands-on experiences activate sensorimotor brain systems that subsequently promote understanding (Kontra et al., 2015), and the neural mechanisms underpinning curiosity and memory (Gruber, Gelman & Ranganath, 2014).

### Delivering information and educational messaging

Educational practitioners and performers have frequently used magic tricks to help boost learning and convey a range of pedagogical messages. Unlike using magic tricks to promote science and mathematics, this work does not necessarily involve revealing the secrets of illusions, but rather uses tricks to energize students, increase attention and communicate key information and ideas. Some writers have speculated that magic tricks are an especially useful tool for teachers as they are entertaining, promote attention and aid knowledge retention (e.g.,  Vidler & Levine, 1981; Frith & Walker, 1983; McCormack, 1985; McCormack, 1990). In addition, several magicians and educational practitioners have employed magic tricks within an educational context, including therapist Sadie Broome (1989) and Broome (1995), ‘The College of Magic’ in South Africa, and magician Kevin Spencer’s ‘Hocus Focus’ initiative (Spencer, 2012). A related strand of work has used magic tricks to deliver specific messages (for instance, those wishing to promote healthy eating might perform a magic trick in which sugary snacks magically transform into vegetables). This type of work has been used to deliver a wide range of messages, including those associated with safety in the workplace (Hoback, 1962), road safety (Arturo, 1951), religion (Hoy, 1956; Laflin, 2000), health (Lustig, 1994), philosophy (Neale & Burger, 1995), organizational behavior (Krell & Dobson, 1999), sales (Kannen, 1998), life-lessons (Bowman, 1986; Bowman, 2002; Bowman, 2004) and environmentalism (Swann, 2019).

Several studies have assessed the use of magic-based interventions within the context of information delivery and educational messaging.

Adipramono & Nindhita (2016) examined the use of magic tricks in English-language teaching to non-native speakers. During the study, a teacher performed a trick and then used the trick as springboard for discussion. Both student feedback and questionnaire responses indicated that the students enjoyed the activity and felt that it helped them to learn vocabulary. In a similar study, Ikhsanudin, Sudarsono & Salam (2019); see also (Ikhsanudin, 2017) had both an English-language teacher and students perform a magic trick, and then use the trick as a topic for discussion. Observational data supported the notion that this activity appeared to boost the students’ levels of engagement, optimism and curiosity.

Spencer (2012) examined the impact of a magic-based intervention in three schools. The study involved students diagnosed with a range of psychological issues, including Autism, Learning Disability and ADHD. Observation checklists, surveys, and informal interviews conducted with both teachers and students suggested that the intervention resulted in several gains, including increased attention, concentration, memory, and motivation. Although encouraging, these results are difficult to fully evaluate due to the lack of detail presented in the report.

Lustig (1994) aimed to dispel myths surrounding AIDS among schoolchildren, and increase their health-related behaviour, via a bespoke magic show. For example, to illustrate the dangers of friends sharing needles, a red handkerchief (representing shared blood) vanished from a prop syringe and re-appeared tied between two white handkerchiefs (representing friends). Post-show questionnaires showed a significant rise in accurate knowledge about AIDS and increased behavioural intent in two important areas (refusing sex and using condoms).

Finally, Moss, Irons & Boland (2017) conducted an online study in which adult participants either (i) watched a video of a magic trick (a gory version of the classic sawing in half trick) and were not told the secret of the illusion, (ii) watched the magic video but were told the secret, (iii) watched a video of a circus act, or (iv) didn’t see any video. Participants then completed the ‘Need For Cognition Scale’ (Cacioppo, Petty & Kao, 1985), watched a neuroscience tutorial video, rated their engagement with the tutorial, and answered questions about its content. Need for cognition was significantly lower among those who saw the magic video and were not told the secret of the illusion. In addition, participants were more engaged with the neuroscience tutorial when they had not seen either the magic or circus videos. There were no differences across the conditions for memory of the tutorial’s content. The authors suggest that watching the magic trick without discovering the secret may have distracted participants and interfered with their ability to focus on the tutorial.

The discrepancy in the results of the latter two studies might have been due to the differences between the magic-based stimuli employed in each study. Whereas Lustig carefully devised tricks that were relevant to the target messaging, the magic video used by Moss et al. was unrelated to the neuroscience tutorial and contained gory footage that may have been inherently distracting.

Future work in this area could resolve this issue by testing the efficacy of magic tricks that are specifically designed to deliver educational messaging, and exploring the attributes of tricks that enhance attention and memory. This latter work could explore a range of factors, including the impact of how easy it is to figure out the secret of the trick, the performing style of the magician presenting the trick, and the emotional impact of the illusion. This work could be experimental in nature, comparing the impact of different types of illusions and presentations with other techniques designed to enhance curiosity and retention. Researchers could also examine how magic-based interventions affect the understanding and recall of material that is both related to the intervention or presented subsequently. It seems likely that the design and delivery of the magic interventions used in this work would benefit from researchers collaborating with knowledgeable magicians. On a methodological level, this work could involve the type of measures often employed to assess attention and memory, including eye tracking, recall tests and the self-report scales used to study boredom (e.g.,  Vodanovich & Watt, 2015). On a more theoretical level, the work has the potential to inform research exploring the relationship between attention, surprise and memory (e.g., Horstmann, 2015), especially as it relates to the perception and impact of seemingly impossible events.

### Boosting critical thinking about the paranormal

The relationship between magic tricks and scepticism has a long history (e.g.,  Hansen, 1985; Hansen, 1992; Truzzi, 1997). The first book in the English language to reveal the secrets behind several magic tricks was written in an attempt to reduce belief in witchcraft (Scot, 1584/1973; Almond, 2011). Similarly, in the late nineteenth and early twentieth century, several high-profile magicians used magic tricks to challenge the claims of psychics and mediums (e.g.,  Maskelyne, 1876; Houdini, 1924). This trend has continued to the present day with, for instance, magician James Randi using magic tricks to expose demonstrations of alleged psychokinetic metal bending (Randi, 1982) and ‘psychological illusionist’ Derren Brown describing some of the trickery underpinning ‘psychic’ readings (Brown, 2007).

A small amount of this work has empirically examined whether magic-based interventions can help to reduce paranormal belief.

Benassi, Singer & Reynolds (1980) had a magician demonstrate several seemingly psychic feats to students. Prior to the performance, the students were told that they were about to see either a genuine psychic, a performer that might be a magician, or a magician. Around a third of those who had been told that they would definitely be watching a magician nevertheless decided that the performer was psychic, prompting the researchers to conclude that magic-based interventions may have a limited impact on those with a strong belief in the paranormal because they tended to ignore disconfirming evidence.

Mohr, Koutrakis & Kuhn (2015) also examined whether observing a magician fake psychic ability affects students’ paranormal beliefs. A performer was introduced as either a magician or a psychic, and then carried out several ‘psychic’ feats. The differing introductions had no impact on an explicit measure of paranormal belief, but introducing the performer as a psychic was associated with higher scores on an implicit measure. A follow-up study failed to replicate these findings (Lesaffre et al., 2018).

Dougherty (2004) also explored the impact of a magic-based critical thinking course on students’ paranormal beliefs. Towards the start of the course, the lecturer used magic tricks to fake paranormal ability and later revealed the secrets behind these tricks. Dougherty reported the findings from two studies, both of which suggested that the course significantly lowered the students’ paranormal beliefs.

Truzzi (1997) discussed whether the use of magic tricks to promote scepticism would be more effective when magicians simply simulated psychic phenomena or also revealed the secrets to their tricks, and argued that revealing secrets was likely to prove counter-productive. The results of the studies reviewed here run contrary to this argument, and suggest that a decline in participants’ paranormal belief is associated with them being told the secrets behind the tricks. This issue could be explored in future research, along with other variables that may affect the impact of magic-based interventions designed to promote scepticism. This work could, for instance, explore the effect of people publically expressing their belief in psychic phenomena prior to seeing a magician duplicate these phenomena, and whether magic-based interventions are equally effective among people with different levels of belief in the paranormal.

### Enhancing creativity in product design

Some studies have shown that solving magic tricks involves divergent thinking (e.g.,  Danek et al., 2014), and there is circumstantial evidence to suggest that watching and/or performing such tricks might help boost creativity. For instance, Subbotsky, Hysted & Jones (2010) showed children film clips that contained either magical content (e.g., talking animals and levitating objects) or non-magical content, and then had them complete a creativity task. The children that had seen the clips with magical content obtained significantly higher creativity scores. In addition, several researchers have suggested that figuring out the solution to a magic trick may help to enhance creative thinking (e.g.,  Vidler & Levine, 1981; McCormack, 1985; McCormack, 1990), and organisations such as London-based ‘Abracademy’ offer magic-based training sessions designed to boost creativity and innovation. However, the only studies to empirically investigate the link between magic tricks and creativity have examined how being exposed to the seemingly impossible effects created by magicians impacts product design.

Some designers have argued that magic tricks often elicit specific positive emotions (e.g., surprise, wonder and delight) and that the principles used to create these illusions could prove helpful in developing products and user experiences (Tognazzini, 1993; Hepworth, 2007). This idea has been tested in studies conducted at the University of Tokyo.

Haritaipan, Saijo & Mougenot (2018a) identified the effects produced by magicians (e.g., appearances, vanishes, levitations, etc.) and then used these concepts to produce a deck of cards and a series of videos. Novice design students were then asked to create several novel ideas for a mug after either studying the cards, viewing the videos or doing neither. These ideas were coded for fluency (number of ideas), flexibility (number of categories of ideas), originality and feasibility. The students seeing the cards and videos produced ideas that were significantly more original and feasible.

Using the same design task, Haritaipan, Saijo & Mougenot (2018b) compared the impact of cards that just described the magical effects (e.g., appearance) with those that also presented methods to achieve those effects (e.g., expansible object, hologram). The findings suggested that the ‘effect plus method’ cards resulted in ideas that were significantly more feasible and flexible.

Although the initial findings from this area appear promising, future work in this area would benefit from using larger cohorts and comparing the magic-based interventions to other techniques commonly employed to boost creativity. This work could also examine the impact of magic-based interventions on more general measures of creativity, such as the Alternative Uses Task, Remote Associates Test and Torrance Tests of Creative Thinking (Said-Metwaly, Kyndt & Van den Noortgate, 2017).

## Conclusions

For hundreds of years, magic tricks have been used within a variety of educational contexts. This work has generated a surprisingly large literature, and has involved magic tricks being used to promote science and mathematics, deliver information and educational messages, boost critical thinking and enhance creativity. However, relatively few studies have assessed the impact of magic tricks on cognition and this work has been often been presented in relatively obscure publications.

As noted throughout this review, much of the work suffers from a variety of methodological issues, including failing to employ control groups, an over-reliance on self-report measures (such as student engagement ratings or belief questionnaires) and assessing the short-term impact of relatively brief interventions. It is hoped that future work in the area will address these shortcomings. One of the most pressing needs is to evaluate the impact of magic-based interventions by comparing their effect to that of a control group or another pedagogical intervention. There exists a large literature on how to develop and test such interventions within educational settings (see, e.g.,  Wagner, 1997; Outhwaite, Gulliford & Pitchford, 2019), and many of the debates and designs within that literature are applicable to the work reviewed in this paper. Second, future researchers might also consider employing ecologically valid variables, including those relating to knowledge retention (e.g., exam results and test scores), attention and engagement (e.g., class attendance, question-asking and eye tracking), attitudinal shifts (e.g., beliefs about science, pseudoscience and the paranormal) and emotional impact (e.g., validated mood questionnaires). Third, the work could also examine the impact of more substantial interventions delivered over longer periods of time. Finally, nearly all of the interventions employed in the studies were developed and delivered by educational practitioners. Conjurers have created a sizable literature on how best to invent and present magic (e.g.,  Ortiz, 1994; Weber, 2004), and future work could explore the possibility of employing this expertise to create more effective interventions.

However, regardless of these shortcomings, almost all of the studies have obtained positive findings and thus suggest that magic tricks have the potential to act as effective educational interventions. If confirmed by future studies, these findings could have both theoretical and practical importance. On a theoretical level, such studies could contribute to several on-going and important debates, including the role of curiosity in learning (e.g.,  Subbotsky, 2010; Pluck & Johnson, 2011), the relationship between experience and belief change (e.g.,  Myers et al., 2013; Mohr, Lesaffre & Kuhn, 2019), why positive and surprising events are highly memorable (e.g.,  Jang et al., 2019; Foster & Keane, 2019), how seemingly mysterious events can be used to promote critical thinking (Blas, 2016), and whether examples of lateral problem solving enhance divergent thinking (Danek et al., 2014).

This work is also likely to have practical implications. For instance, educational practitioners interested in the notion of ‘serious fun’ have long explored how learning is enhanced by activities that are enjoyable, interactive and hands-on (e.g.,  Schulz & Bonawitz, 2007; Masterson & Bohart, 2019). Most of this work has focused on activities that enjoy widespread appeal (such as structured play, games and drama) and tends to overlook magic-based interventions. This is unfortunate as magic tricks embody the key attributes associated with ‘serious fun’, and can be performed with everyday objects in almost any setting. As such, they have the potential to form the basis for practical and cost-effective interventions in a variety of educational contexts.

In short, research suggests that magic-based interventions are effective in a range of educational contexts, including communicating science, mathematics and computing, delivering information and educational messages, and promoting both critical and creative thinking. However, this body of work suffers from several methodological shortcomings, including a lack of control groups, an over reliance on self-report measures and a focus on the short-term impact of relatively brief interventions. As such, it mirrors many of the issues associated with the use of magic-based interventions to promote physical and psychological wellbeing (Wiseman & Watt, 2018; Bagienski & Kuhn, 2019). It is hoped that future research in the area will address these issues, and will help researchers and practitioners to explore the benefits of thinking about the impossible.

## Additional Information and Declarations

### Data Availability

The following information was supplied regarding data availability: This is a literature review article and did not generate raw data.
